# Supplementary material for: Metabolic Alteration of Trypanosoma cruzi during Differentiation of Epimastigote to Trypomastigote Forms
Source: Pathogens. 2022 Feb 19;11(2):268. doi: 10.3390/pathogens11020268 (PMC8879499; doi:10.3390/pathogens11020268)
Supplement: Supplementary file 1 [file pathogens-11-00268-s001.zip › pathogens-1538742-supplementary.pdf]

Supplementary Table S1 - Concentration of organic acids in different times of the differentiation of the *Trypanosoma cruzi* in TAU3AAG medium, obtained through the Aminex HPX 87H column.

Organic acids	24 h	48 h	72 h
Glicerate-2-P	1890.02 ± 132.1	1547.12 ± 82. 5	811.62 ± 38.76
Citrate	24.12 ± 1.51	31.08 ± 1.77	8.89 ± 4.34
Piruvate	357.04 ± 17.7	206.23 ± 14.5	10.12 ± 8.43
Malate	10.01 ± 0.54	27.02 ± 2.65	20.43 ± 7.12
Succinate	27.30 ± 1.91	226.12 ± 13.61	199.66 ± 8.34
Acetate	381.43 ± 29.35	342.03 ± 21.44	259.24 ± 23.45

The values corresponds the media ± SD of the concentration in nmoles of organic acid of three experiments realized with a population of ~$3x10^8$ parasites.

Supplementary Table S2 - Concentration of the acids organic during the growth of the *Trypanosoma cruzi* in BHI medium, obtained through the Aminex HPX 87H column.

Organic Acid	24 h	72 h	12h	168 h
Glicerate-2-P	1695.5 ± 92.2	1569.3 ± 6.31	1809.2 ± 93.62	1484.2 ± 121.11
Citrate	129.3 ± 9.11	169.2 ± 9.46	326.4 ± 11.31	281.3 ± 28.01
Piruvate	2.4 ± 0.11	1.7 ± 0.03	2.7 ± 0.80	4.7 ± 1.10
Malate	2.6 ± 0.12	2.7 ± 0.11	0.7 ± 0.05	0.6 ± 0.03
Succinate	2.2 ± 0.07	3.5 ± 0.15	4.2 ± 0.41	2.9 ± 0.08
Acetate	59.1 ± 3.13	102 .2 ± 3.78	185.5 ± 12.21	147.2 ± 7.60

The values correspond the media ± SD of the concentration in nmoles of organic acid of three experiments realized with a population of ~$3x10^8$ parasites.

Supplementary Table S3- Correlation between the percentage of epimastigote and tripomastigote forms and the specific enzymatic activity of aldolase (Ald), hexokinase (Hk) and piruvate kinase (Pk) enzymes during the differentiation of the *T. cruzi* in TAU3AAG medium.

Time	%	%	Specific Activity		
(h)	trypo	epi	Pk	Hk	Ald
0	<1	>100	0.11 ±0.02	0.30 ±0.00	4.40 ±0.70
24	25	75	0.08 ±0.01	0.23 ±0.04	2.00 ±0.20
48	50	50	0.01 ±0.00	0.03 ±0.01	0.34 ±0.00
72	70	30	0.06 ±0.00	0.11 ±0.02	0.74 ±0.02

The values of specific activity correspond the average with the standard deviation of three experiments accomplished in copy. The unit for Pk and Hk was μmoles subst/min/mg protein and for the aldolase was nmoles subst/min/mg protein. ~$2 x 10^8$ parasites were used in each assay.

Supplementary Table S4- Specific enzymatic activity of the aldolase (Ald), hexokinase (Hk) and piruvate kinase (Pk) extracted from epimastigotes maintained in BHI medium.

**Time**	**Specific Activity**		
**(h)**	**Pk**	**Hk**	**Ald**
**24**	**0.02 ±0.09**	**0.09 ±0.03**	**3.97 ±0.03**
**72**	**0.08 ±0.03**	**0.23 ±0.01**	**4.04 ±0.25**
**120**	**0.10 ±0.02**	**0.29 ±0.00**	**4.05 ±0.02**

The values of specific activity correspond the average with the standard deviation of two experiments accomplished in copy. The unit for Pk and Hk was μmoles subst/min/mg protein and for the aldolase was nmoles subst/min/mg protein. ~2 x 10^8 parasites were used in each experiment of enzymatic determination.

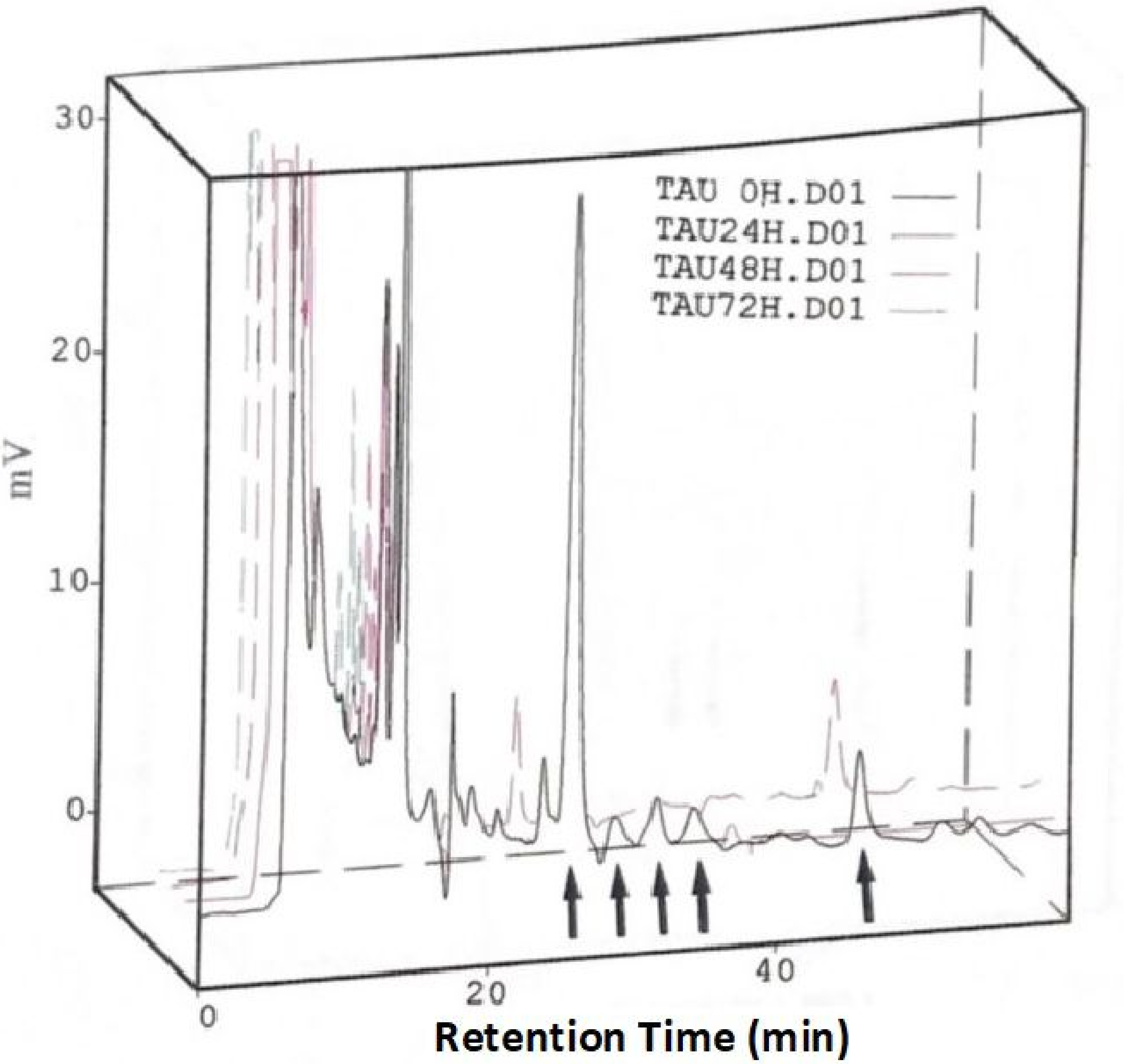
30
20
10
0
mV
TAU 0H.D01
TAU24H.D01
TAU48H.D01
TAU72H.D01
0
20
40
Retention Time (min)

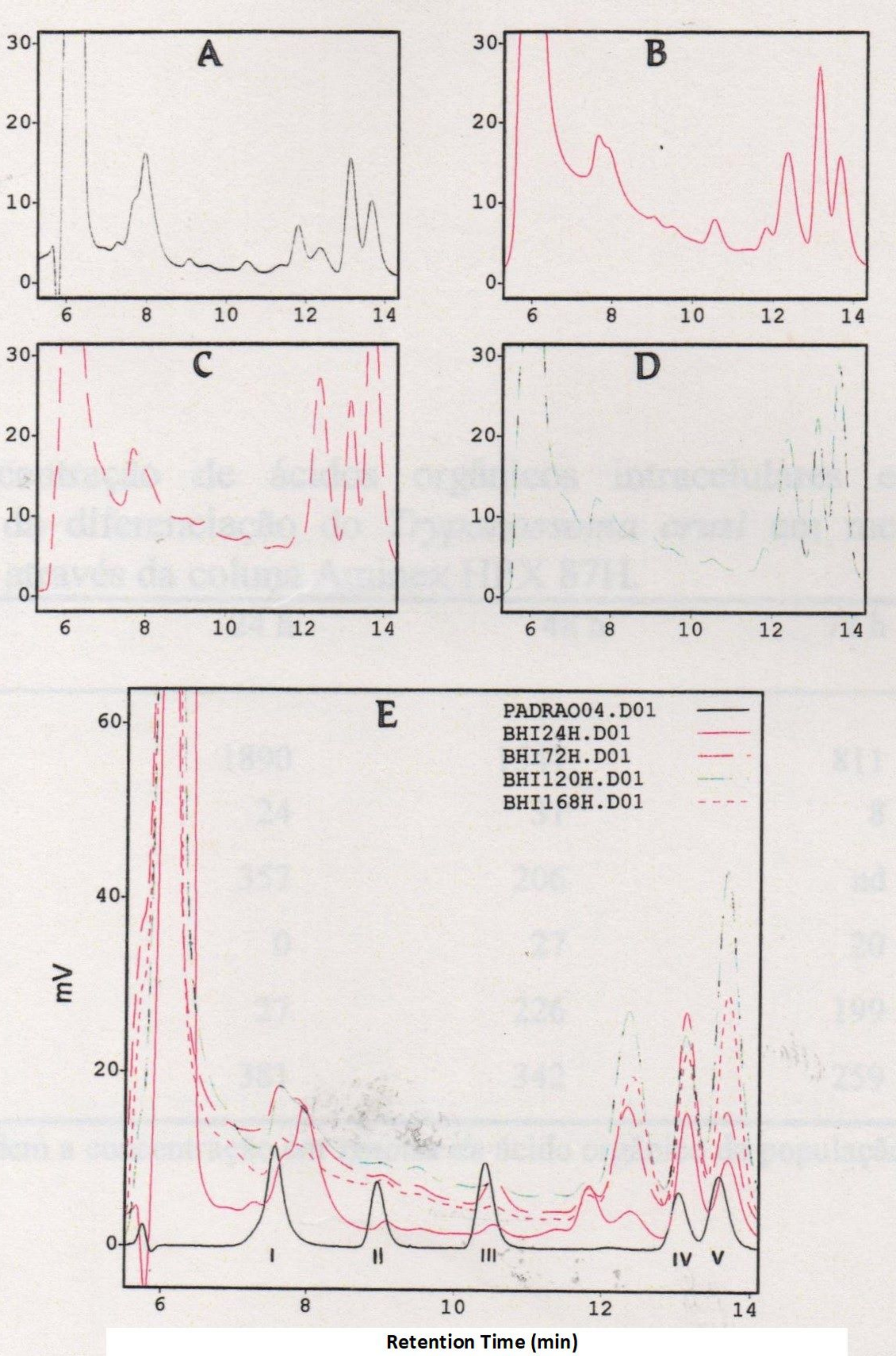
A
B
C
D
E
PADRAO04.D01
BHI24H.D01
BHI72H.D01
BHI120H.D01
BHI168H.D01
mV
I
II
III
IV
V
Retention Time (min)
